# Endoscopic treatment of a hepatic abscess arising from a biliary stricture due to intraductal papillary neoplasm of the bile duct

**DOI:** 10.1055/a-2253-0948

**Published:** 2024-03-08

**Authors:** Kamal M. Hassan, Enad Dawod, Mohammed Hassan, Sanjay M. Salgado, SriHari Mahadev, Reem Z. Sharaiha, Kartik Sampath

**Affiliations:** 1 12295 Gastroenterology and Hepatology, Weill Cornell Medical College, New York, United States; 2 386498 Alivation, Lincoln, United States

Endoscopic ultrasound (EUS)-guided drainage, primarily used for intra-abdominal abscesses/fluid collections, is increasingly used for hepatic abscesses that have been traditionally managed by external drainage or surgery. EUS interventions offer reduced morbidity and potentially lower complication rates. We present a case showcasing the efficacy and safety of EUS-guided drainage in treating a complex hepatic abscess ( Video 1 ).

Endoscopic management of a large hepatic abscess secondary to a severe chronic biliary stricture due to intraductal papillary neoplasm of the bile duct.Video 1

**Fig. 1:**
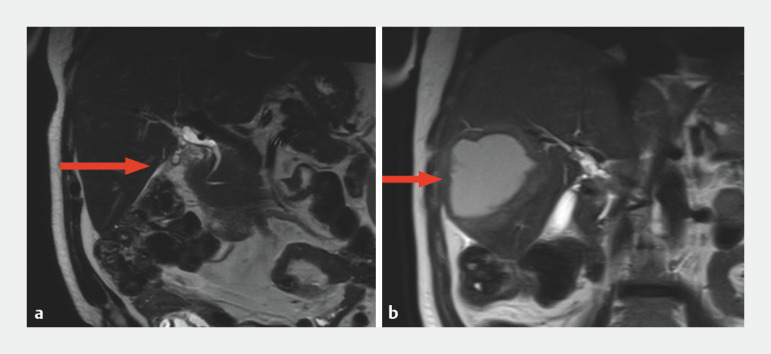
Magnetic resonance cholangiopancreatography images showing: **a** mild-to-moderate intrahepatic biliary dilatation in liver segment 5; **b** an 8.8-cm thick-walled cystic lesion in the right hepatic lobe near the previously dilated biliary segment 4 months after ERCP and sphincterotomy.

A 63-year-old man with abdominal pain and elevated alkaline phosphatase was found to have a dilated right-sided bile duct and mild-to-moderate intrahepatic biliary dilatation with stones ( Fig. 1 **a** ). After an endoscopic retrograde cholangiopancreatography (ERCP) with biliary sphincterotomy and stent placement had been performed, a follow-up ERCP revealed a benign-appearing biliary stenosis, with brush cytology showing benign epithelial cells. Four months later, the patient experienced worsening symptoms. A further scan revealed an hepatic abscess in segment 6 ( Fig. 1 **b** ). Another ERCP confirmed a biliary stricture with prestenotic dilatation and debris in the dilated hepatic duct. Attempts to access the cavity via the transpapillary route failed, leading us to a planned EUS-guided drainage.

**Fig. 2:**
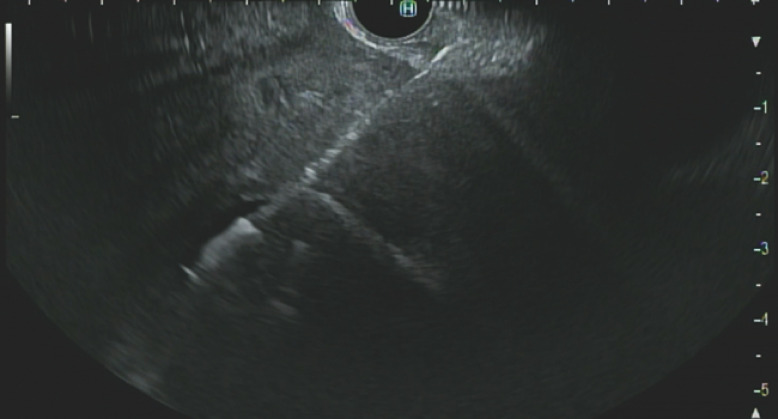
Endoscopic ultrasound (EUS) image during EUS-guided cystoduodenostomy.

**Fig. 3:**
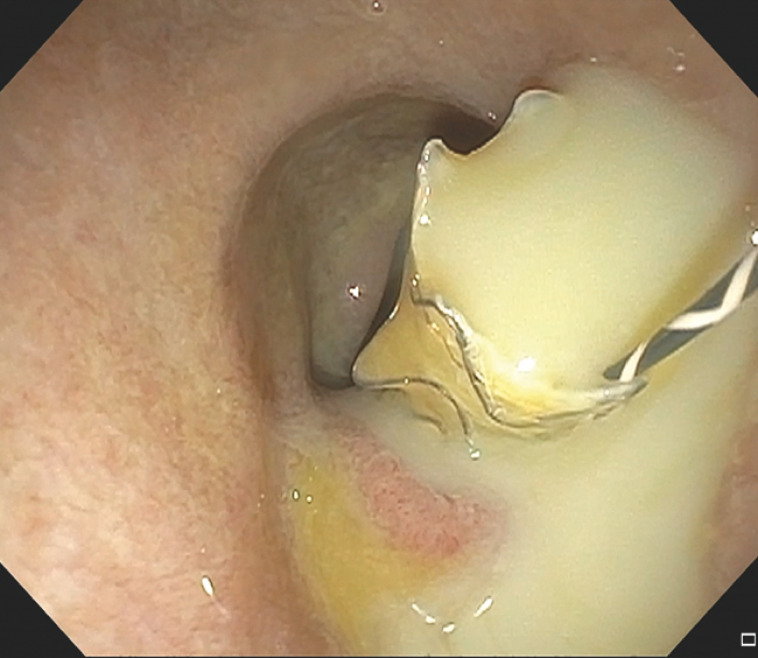
Endoscopic image showing copious pus draining from cystoduodenostomy tract after placement of a fully covered metal stent.

EUS-guided fine-needle aspiration (FNA) confirmed the presence of pus in the abscess, and EUS-guided cystoduodenostomy was performed ( Fig. 2 ). A 10-mm×10-cm metal stent and a 7-Fr×15-cm double-pigtail plastic stent were placed, enabling significant pus drainage ( Fig. 3 ). Both the biliary and cystoduodenostomy stents were correctly placed, and this was confirmed by fluoroscopy. The patient was monitored post-procedure on a liquid to low-residue diet progression, with antibiotics prescribed.

**Fig. 4:**
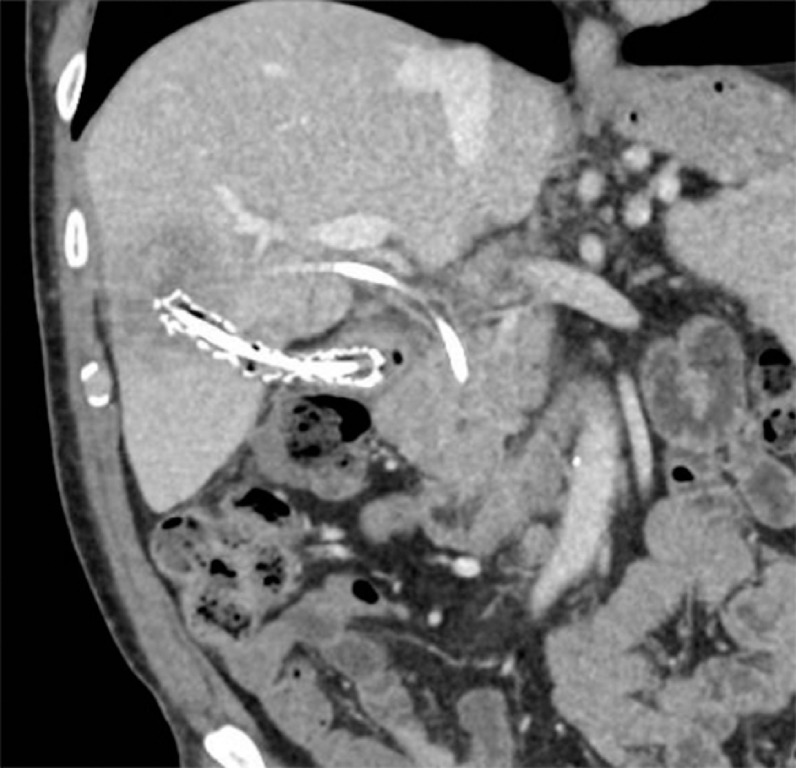
Follow-up computed tomography scan 1 month after the procedure showing abscess resolution and reduction in the biliary dilatation.

A follow-up computed tomography (CT) scan 1 month later showed resolution of the abscess and decreased biliary dilatation ( Fig. 4 ). The cystoduodenostomy stents were removed, while the biliary stent was left in place. After review by the multidisciplinary team, the patient underwent robot-assisted right hepatectomy, which revealed an intraductal papillary neoplasm of the bile duct with high grade dysplasia. The patient showed significant symptom improvement post-surgery.

In conclusion, chronic biliary strictures can lead to cholangitis and hepatic abscesses. EUS-guided drainage is a safe and effective approach, which may be useful alone or with ERCP, for managing liver abscesses and as a bridge to surgery in complex biliary conditions.

Endoscopy_UCTN_Code_TTT_1AR_2AG

